# Association between arthritis and cardiovascular risk factors in community-based adults: an opportunity to target cardiovascular risk

**DOI:** 10.1186/s12872-022-02674-x

**Published:** 2022-05-19

**Authors:** Julia Sewell, Sultana Monira Hussain, Yuanyuan Wang, Anita E. Wluka, Yuan Z. Lim, Melinda J. Carrington, Katherine Samaras, Flavia M. Cicuttini

**Affiliations:** 1 Department of Epidemiology and Preventive Medicine, School of Public Health and Preventive Medicine, Monash University, 553 St Kilda Road, Melbourne, VIC 3004 Australia; 2 Pre-Clinical Disease and Prevention, Baker Heart and Diabetes Institute, 75 Commercial Rd, Melbourne, VIC 3004 Australia; 3 Clinical Obesity, Nutrition and Adipose Biology Laboratory, Healthy Ageing, Garvan Institute of Medical Research, 384 Victoria St, Darlinghurst, NSW 2010 Australia; 4 Department of Endocrinology, St Vincent’s Hospital, Victoria St, Darlinghurst, NSW 2010 Australia; 5 St Vincent’s Clinnical School, University of New South Wales Sydney, Victoria St, Darlinghurst, NSW 2010 Australia

**Keywords:** Arthritis, Osteoarthritis, Inflammatory arthritis, Crystal arthritis, Cardiovascular disease, Cardiovascular risk factors, Obesity, Hypertension, Diabetes, Dyslipidaemia

## Abstract

**Background:**

Undertreated risk factors are major contributors to the burden of cardiovascular disease (CVD). Those with arthritis have an increased prevalence of CVD risk factors. CVD risk factors are often asymptomatic, which may be a barrier their treatment. Arthritis causes pain and immobility, and is a common reason for individuals to seek healthcare. Our aims were to (1) examine the relationship between arthritis and CVD risk factors in Australian adults, and (2) calculate the proportion of CVD risk factors that could be reduced if individuals with arthritis were targeted.

**Methods:**

This cross-sectional study uses data from the 2017–18 Australian National Health Survey which included 13,776 participants, categorised into young (18–39 years), middle aged (40–64 years) and older (≥ 65 years) adults. Hypertension, height and weight were measured. Arthritis, dyslipidemia and diabetes were self-reported. The associations between arthritis and CVD risk factors were examined using logistic regression, and the population attributable fraction (PAF) of arthritis for each CVD risk factor was calculated.

**Results:**

Arthritis was reported by 4.0% of young adults, 28.8% of middle-aged adults and 54.5% of older adults. Those with arthritis were at increased odds of obesity (2.07 fold in young, 1.75 fold in middle-aged and 1.89 fold in older adults), increased odds of diabetes (5.70 fold in young, 1.64 fold in middle-aged and 1.37 fold in older adults), increased odds of hypertension (2.72 fold in young, 1.78 fold in middle-aged and 1.48 fold in older adults) and an increased odds of dyslipidaemia (4.64 fold in young, 2.14 fold in middle-aged and 1.22 fold in older adults) compared to those without arthritis. This elevated chance remained significant even after adjusting for obesity, with the exception of diabetes in the older population. This elevated chance remained significant even after adjusting for obesity, with the exception of diabetes in the older population. The PAF of the presence of arthritis for having at least one CVD risk factor was 30.7% in middle-aged adults and 70.4% in older adults.

**Conclusion:**

Australian adults of all ages with arthritis are at increased odds of having CVD risk factors. For young and middle-aged adults, this increased odds remains significant even when adjusted for obesity. Presentation to healthcare practitioners with arthritis is an opportunity to screen for asymptomatic CVD risk factors with the potential of improving outcomes for both diseases. By adopting an approach of managing arthritis and CVD risk factors in parallel, rather than in silos, we could reduce the burden of CVD risk factors by 20–30%.

## Background

Cardiovascular diseases (CVD) remain the leading cause of disease burden in the world [1]. CVD burden has continued to rise for decades in almost all countries, and alarmingly the age-standardized rate of CVD has begun to rise. According to the Global Burden of Disease study, CVD was the underlying cause of 6.2 million deaths occurring between the ages of 30 and 70 years, and one-third of all deaths globally in 2019 [2]. The underlying pathophysiology of CVD is accelerated by obesity, hypertension, dyslipidemia and diabetes [3]. These conditions develop over many years, remaining silent, and thus are usually advanced by the time CVD symptoms occur [4]. Despite traditional risk score screening being recommended in guidelines for identifying individuals at risk of developing CVD [4–7], this remains underutilised [8]. Addressing these risk factors in all affected individuals is challenging, as many people with these CVD risk factors are largely asymptomatic and may not be actively seeking healthcare to specifically address their CVD risk. Hence, using other risk-enhancing factors to identify subsets of people most likely to receive clinical benefit for prevention of CVD offers an alternative approach.

Musculoskeletal conditions such as arthritis, in contrast, cause pain and immobility and are one of the most common reasons for presentation to primary healthcare [9]. People with most forms of arthritis (e.g. inflammatory arthritis, gout and osteoarthritis or OA) have an increased risk of CVD and death from CVD [10–14]. For example, the rate of CVD is increased by 1.5–2.0 fold in those with rheumatoid arthritis [15], 1.3 fold in systemic connective tissue disorders [16], 1.5 fold in gout [17] and twofold in OA [18]. Shared inflammatory pathways has been suggested as a possible link for any form of these arthritis and CVD. Therefore presentations for arthritis symptoms may be used as a ‘teachable moment’, or an opportunity for healthcare practitioners to manage arthritis and screen for CVD risk factors in parallel in order to reduce the burden of both conditions. Thus, our aim was to examine the relationship between arthritis and CVD risk factors (obesity, hypertension, dyslipidaemia and diabetes) in young, middle-aged and older Australian adults and to calculate the proportion of individuals with CVD risk factors in the population that could be reduced by targeting arthritis.

## Methods

### Study design

This is a cross-sectional study using the National Health Survey (NHS) data conducted by the Australian Bureau of Statistics (ABS) in 2017–18 [19].The NHS was conducted in metropolitan, regional and rural areas of all Australian states and territories. Participants were excluded if they were under the age of 18 since both OA and CVD are less common in this age category [18].

### Demographic, anthropometric and clinical measurement

Trained ABS interviewers conducted interviews between 2 July 2017 and 30 June 2018. Voluntary measures of height and weight were collected from respondents. If respondents elected not to be measured, they were asked to self-report their height and weight. In total 80% of respondents agreed to be measured and the remainder elected to self-report these data [20]. Body mass index (BMI) was calculated using the formula weight (kg) divided by the square of height (m), and obesity was defined by BMI > 30 kg/m^2^. Voluntary blood pressure measurements were also taken. The second of two readings was counted, unless there was a difference of > 10 mmHg between the two readings in which case a third reading was taken [19]. Hypertension was defined by a systolic blood pressure of > 140 mmHg or a diastolic blood pressure of > 90 mmHg [21].

Respondents were asked whether they had arthritis, including osteoarthritis, rheumatoid arthritis, rheumatism, gout or other types of arthritis [19]. The presence of arthritis was defined if the condition had been diagnosed by a doctor or a nurse, or if it was a current or long-term condition [19]. Respondents were also asked if they had been diagnosed with any type of diabetes or high blood sugar by a doctor or a nurse and were included as having diabetes if they responded yes, except if they had gestational diabetes [19]. Similarly, respondents were asked whether they had ever been told by a doctor or nurse if they had high cholesterol [19].

### Statistical analysis

Study participants were categorised into three age groups: young (18–39 years old), middle-aged (40–64 years old), and older (65 years old and above) adults. Descriptive statistics were used to describe the population characteristics and distribution of arthritis and CVD risk factors. Logistic regression models were used to estimate the odds ratio (OR) with 95% confidence intervals (CI) for CVD risk factors in relation to the presence of arthritis. Population attributable fraction (PAF) was calculated to determine the proportion of CVD risk factors in the population that could be attributable to having arthritis using the “punafcc” command in Stata, which implements the method recommended by Greenland and Drescher [22]. *P* values less than 0.05 were considered statistically significant. Analyses were adjusted for obesity > 30 kg/m^2^. All analyses were performed using STATA 15.0 SE (StataCorp LP., College Station, TX, USA).

## Results

Our study included 13,776 participants. Arthritis was reported by 4.0% of young adults, 28.8% of middle-aged adults and 54.5% of older adults. Those with arthritis were at increased odds of obesity (2.07 fold in young, 1.75 fold in middle-aged and 1.89 fold in older adults), increased odds of diabetes (5.70 fold in young, 1.64 fold in middle-aged and 1.37 fold in older adults), increased odds of hypertension (2.72 fold in young, 1.78 fold in middle-aged and 1.48 fold in older adults) and an increased odds of dyslipidaemia (4.64 fold in young, 2.14 fold in middle-aged and 1.22 fold in older adults) compared to those without arthritis. This elevated odds remained significant even after adjusting for obesity, with the exception of diabetes in the older population.

### Young adults (18–39 years)

The prevalence, associations and PAF of CVD risk factors and arthritis in young adults are presented in Table 1. Of this population, 29.1% of those with arthritis were obese, compared to 16.5% of those without arthritis, indicating a 2.07 fold higher prevalence (95% CI 1.36–3.16) of obesity in young adults with arthritis. The PAF, or the proportion of the young adult population with obesity that could be attributable to arthritis, was 15.0% (95% CI 4.1–24.7%). The prevalence of diabetes was 2.5% in young adult with arthritis; a 5.70 fold higher prevalence (95% CI 1.74–15.37) compared to those without arthritis, which remained significant after adjusting for obesity. The prevalence of hypertension and dyslipidaemia in young adult with arthritis was 8.9% for each, a 2.72 (95% CI 1.53–4.84) and 4.64 fold (95% CI 2.56–8.39) higher prevalence compared to those without arthritis, respectively. These results remained significant after adjusting for obesity. The PAF for hypertension and dyslipidemia in relation to arthritis were 5.6% (95% CI 0.9–10.2%), and 7.0% (95% CI 2.3–11.5%), respectively. In young adult with arthritis, 13.4% had at least one of hypertension, dyslipidaemia or diabetes compared to 5.2% of those without arthritis, a 2.82 fold (95% CI 1.74–4.56) higher prevalence and a PAF of 8.6% (95% CI 2.7–14.1%). If obesity was included, 36.4% of this population with arthritis had at least one CVD risk factor (hypertension, dyslipidaemia, diabetes or obesity), compared to 20.6% of those without arthritis, a 2.28 fold (95% CI 1.53–3.40) higher prevalence and a PAF of 20.4% (95% CI 8.2–31.0%).

**Table 1:** Cardiovascular risk factors in adults with/without arthritis aged 18–39 years in NHS 2017–18

	Total number		OR (95% CI)		Population attributable fraction, % (95% CI)
	No arthritis (n = 3773)	Arthritis (n = 157)	OR (95% CI)	OR adjusted for obesity (95% CI)	
Obesity^a^	473 (16.5%)	32 (29.1%)	2.07 (1.36–3.16)	–	15.0 (4.1–24.7)
Diabetes	19 (0.5%)	4 (2.5%)	5.70 (1.74–15.37)	4.87 (1.34–17.69)	2.1 (0.00–4.50)
Hypertension	131 (3.5%)	14 (8.9%)	2.72 (1.53–4.84)	2.35 (1.17–4.70)	5.6 (0.9–10.2)
Dyslipidaemia	78 (2.1%)	14 (8.9%)	4.64 (2.56–8.39)	4.62 (2.34–9.14)	7.0 (2.3–11.5)
1 or more of hypertension/dyslipidaemia/diabetes	196 (5.2%)	21 (13.4%)	2.82 (1.74–4.56)	2.78 (1.59- 4.88)	8.6 (2.7–14.1)
1 or more of hypertension/dyslipidaemia/diabetes/obesity^a^	613 (20.6%)	40 (36.4%)	2.28 (1.53–3.40)	–	20.4 (8.2–31.0)

^a^Obesity data available n = 2971

### Middle-aged adults (40–64 years)

The prevalence, associations and PAF of CVD risk factors and arthritis in middle-aged adults are presented in Table 2. The prevalence of obesity was 38.3% in those with arthritis compared to 26.2% in those without, a 1.75 fold (95% CI 1.54–2.01) higher prevalence. The PAF of arthritis for obesity was 16.5% (95% CI 12.5–20.3%). The prevalence of diabetes was 9.2% in middle-aged adults with arthritis, representing a 1.64 fold (95% CI 1.33–2.03) higher prevalence compared to those without arthritis, remaining significant after adjusting for obesity. The PAF of arthritis for diabetes was 3.6% (95% CI 1.9–5.3%). The prevalence of hypertension and dyslipidaemia in this population with arthritis was 28.6% and 21.9% respectively, with a 1.78 fold (95% CI 1.60–2.04) and 2.14 fold (95% CI 1.84–2.49) higher prevalence compared to those without arthritis, remaining significant after adjusting for obesity. The PAF of arthritis was 5.6% (95% CI 0.8–10.2%) for hypertension and 11.6% (95% CI 9.1–14.0%) for dyslipidaemia. Among middle-aged adults, 41.5% of those with arthritis had at least one of hypertension, dyslipidaemia or diabetes, a 1.90 fold (95% CI 1.69–2.15) higher prevalence and a PAF of 19.7% (95% CI 16.0–23.2%). If obesity was included, 60.4% of this population with arthritis had at least one of hypertension, diabetes, dyslipidaemia or obesity, a 2.03 fold (95% CI 1.79–2.31) higher prevalence and a PAF of 30.7% (95% CI 25.5–35.5%).

**Table 2:** Cardiovascular risk factors in adults with/without arthritis aged 40–64 years in NHS 2017–18

	Total number		OR (95% CI)		Population attributable fraction, % (95% CI)
	No arthritis (n = 4055)	Arthritis (n = 1638)	OR (95% CI)	OR adjusted for obesity (95% CI)	
Obesity^a^	868 (26.2%)	527 (38.3%)	1.75 (1.54–2.01)	–	16.5 (12.5–20.3)
Diabetes	236 (5.8%)	151 (9.2%)	1.64 (1.33–2.03)	1.37 (1.08–1.73)	3.6 (1.9–5.3)
Hypertension	745 (18.4%)	496 (28.6%)	1.78 (1.60–2.04)	1.59 (1.37–1.84)	5.6 (0.8–10.2)
Dyslipidaemia	469 (11.6%)	358 (21.9%)	2.14 (1.84–2.49)	2.04 (1.73–2.41)	11.6 (9.1–14.0)
1 or more of hypertension/dyslipidaemia/diabetes	1101 (27.2%)	680 (41.5%)	1.90 (1.69–2.15)	1.73 (1.51–1.98)	19.7 (16.0–23.2)
1 or more of hypertension/dyslipidaemia/diabetes/obesity^a^	1421 (42.8%)	830 (60.4%)	2.03 (1.79–2.31)	–	30.7 (25.5–35.5)

^a^Obesity data available n = 4694

### Older adults (> 65 years)

The prevalence, associations and PAF of CVD risk factors and arthritis in older adults are presented in Table 3. The prevalence of obesity was 32.9% among those with arthritis, a 1.89 fold (95% CI 1.62–2.21) higher prevalence compared to those without arthritis. The PAF was 15.5% (95% CI 12.0–18.9%). The prevalence of diabetes in those with arthritis was 17.6%, which represents a 1.37 fold (95% CI 1.15–1.62) increased prevalence compared to those without arthritis. The PAF of arthritis for diabetes was 4.7% (95% CI 2.2–7.2%). The prevalence of hypertension and dyslipidaemia in this population with arthritis were 48.2% and 28.7% respectively, a 1.48 fold (95% CI 1.31–1.68) and 1.22 fold (95% CI 1.07–1.41) increased prevalence respectively compared to those without arthritis, which remained significant when adjusted for obesity. The PAF was 15.7% (95% CI 11.0–20.1%) for hypertension and 5.2% (95% CI 1.7–8.7%) for dylipidemia. 61.7% of older adults with arthritis had at least one of hypertension, dyslipidaemia or diabetes, representing a 1.49 fold (95% CI 1.31–1.68) higher prevalence compared to those without arthritis and a PAF of 20.2% (95% CI 14.4–25.6%). Of older adults with arthritis, 70.4% had at least one of hypertension, dyslipidaemia, diabetes or obesity, which equates to a 1.58 fold (95% CI 1.38–1.83) higher prevalence compared to those without arthritis and a PAF of 26.0% (95% CI 18.8–32.5%).

**Table 3:** Cardiovascular risk factors in adults with/without arthritis aged ≥ 65 years in NHS 2017–18

	Total number		OR (95% CI)		Population attributable fraction, % (95% CI)
	No arthritis (n = 1891)	Arthritis (n = 2262)	OR (95% CI)	OR adjusted for obesity (95% CI)	
Obesity^a^	326 (20.6%)	610 (32.9%)	1.89 (1.62–2.21)	–	15.5 (12.0–18.9)
Diabetes	256 (13.5%)	399 (17.6%)	1.37 (1.15–1.62)	1.15 (0.95–1.39)	4.7 (2.2–7.2)
Hypertension	730 (38.6%)	1091 (48.2%)	1.48 (1.31–1.68)	1.35 (1.18–1.55)	15.7 (11.0–20.1)
Dyslipidaemia	468 (24.8%)	649 (28.7%)	1.22 (1.07–1.41)	1.19 (1.02–1.38)	5.2 (1.7–8.7)
1 or more of hypertension/dyslipidaemia/diabetes	984 (52.0%)	1396 (61.7%)	1.49 (1.31–1.68)	1.37 (1.19–1.57)	20.2 (14.4–25.6)
1 or more of hypertension/dyslipidaemia/diabetes/obesity^a^	948 (60.0%)	1303 (70.4%)	1.58 (1.38–1.83)	–	26.0 (18.8–32.5)

^a^Obesity data available n = 343

## Discussion

This study showed high prevalences of treatable CVD risk factors in people with arthritis. The proportion of one or more CVD risk factor (hypertension, dyslipidaemia or diabetes) in the population that could be identified by targeting those with arthritis was 8.6% in young adults, 19.7% in middle-aged adults and 20.2% in older adults. These proportions were significantly higher if obesity was included, being 20.4%, 30.7%, and 26.0%, respectively.

The prevalences of arthritis across each age group is similar to those reported in other Australian literature [23, 24] and in other Western countries [25, 26]. Although the type of arthritis was not specified in our study, it is likely to vary across each age group. However, there is evidence that all the common forms of arthritis; osteoarthritis [27, 28], inflammatory arthritis [29–31] and crystal arthritis such as gout [32, 33], are associated with an increased risk of CVD, which is significantly attributed to the traditional risk factors such as hypertension and dyslipidaemia.

CVD remains the number one cause of death worldwide, with significant burden of disease due to untreated or undertreated risk factors [34]. In Australia, it was found that 68% of people with hypertension were either not treated or undertreated [35], contributing to 48% of CVD burden [36]. Approximately 80% of Australians with dyslipidaemia are not on treatment [20], contributing to 21% of CVD burden [36] while obesity is estimated to contribute to approximately 30% of CVD burden [36]. Only 55% of Australians with diabetes are well-controlled, attaining target haemoglobin A1c levels of less than 7.0% [35]. One barrier to management of CVD risk factors is that they are mostly asymptomatic and thus require specific targeting approaches. As seen in our study, it has been noted over a number of years that those with arthritis have an increased prevalence of CVD risk factors [28].

Arthritis, in contrast to CVD risk factors, causes pain and immobility and is a very common reason to seek health care [9]. Given that CVD risk factors are common in those with all forms of arthritis, and that those with arthritis are more likely contact with health professionals, this point of contact has the potential to be used as a teachable moment to signal the assessment of CVD risk. This may also be a strategy to aid in targeting higher risk individuals who are less likely to seek medical attention for preventative (asymptomatic) care [37–41]. The potential to target the hidden burden of CVD risk would benefit those with arthritis. Further, given the high prevalence of arthritis this has the potential to impact on the overall burden of CVD. We estimate that targeting those with arthritis has the potential to identify 5.6% of hypertension in young and middle-aged adults and 15.7% in older adults in these populations. Similarly, by targeting those with arthritis, there is the potential to identify 7.0% of dyslipidemia in young adults, 11.6% in middle-aged adults and 5.2% in older adults in these populations.

The prevalence of arthritis is high in the community, and arthritis symptoms are a common reason to seek healthcare attention [9]. Finding people with arthritis and screening them for CVD presents an opportunity for improving community-based CVD prevention as cardiovascular risk factors are asymptomatic until CVD develops. The current study shows that there is the opportunity to target people for CVD prevention at their first point of contact for joint pain. This has the potential to increase awareness of cardiovascular prevention by targeting a population with a high prevalence of CVD risk factors. This approach in turn also has the potential of reducing joint pain since CVD and OA, the most common cause of joint pain in adults, share common risk factors and we have shown that targeting these through low-intensity lifestyle intervention improves joint pain [42].

The results of this study need to be considered in context of its limitations. While hypertension and obesity were mostly defined by measurements, the prevalence of dyslipidemia and diabetes was self-reported, which may have been subject to bias and resulted in underestimation. As an example, in the NHS 2017–18 it was estimated that nearly three quarters (73.7%) of all adults with measured high blood pressure did not report having hypertension [43]. Our results may be limited by the use of dichotomous variables (i.e. the presence or absence) of conditions. There were no supporting clinical, biochemical or radiographic data which not only would confirm diagnoses, but could also be used to grade severity. There may be a stronger association between more severe CVD risk factors and arthritis, which would not be elucidated in our study. The type of arthritis was not specified in our data. While it may be possible to infer that in the younger population inflammatory arthritis may predominate, as with OA in the older population, the types of arthritis are separate entities and may cause an increased CVD risk via different pathophysiological processes. Since the association between inflammatory arthritis (eg. rheumatoid arthritis, gout) and CVD are increasingly recognised, people with these conditions may have been more likely to be screened for CVD risk factors thereby possibly introducing bias. However, the association between OA and CVD risk factors is not well recognised among healthcare professionals and specific CVD screening is not recommended in this population in either CVD screening guidelines or in guidelines for the management of OA. As the prevalence of inflammatory arthritis is far less common than osteoarthritis in middle aged and older populations, it is unlikely that bias due to screening for CVD risk factors is the only explanation for our findings as arthritis was associated with elevated CVD risk irrespective of age category. Another limitation is that we did not have access to data pertaining to physical activity levels, which could act as a potential confounding factor in some of the relationships drawn. Finally, this is a cross-sectional study, thus a temporal relationship between arthritis and risk factors of CVD could not be established. This study can confirm that both arthritis and CVD coexists and if one of these health risk is present, people should be referred to test the other condition.

## Conclusions

Our study demonstrates that Australian adults of all ages with arthritis are at an increased odds of having CVD risk factors independent of obesity. Presentation to healthcare practitioners with symptoms due to arthritis could provide an opportunity to screen for individuals with asymptomatic CVD risk factors. By adopting an approach of managing arthritis and CVD risk factors in parallel, rather than in silos, there is the potential to improve outcomes in both conditions.

## Data Availability

The datasets used and/or analysed during the current study are available from the corresponding author on reasonable request.

## Abbreviations

CVD: Cardiovascular disease
PAF: Population attributable fraction
OA: Osteoarthritis
NHS: National Health Survey
ABS: Australian Bureau of Statistics
BMI: Body mass index
OR: Odds ratio
CI: Confidence interval
